# A brief review on resistance to P2Y_12_ receptor antagonism in coronary artery disease

**DOI:** 10.1186/s12959-019-0197-5

**Published:** 2019-05-20

**Authors:** Ellen M. K. Warlo, Harald Arnesen, Ingebjørg Seljeflot

**Affiliations:** 1 Center for Clinical Heart Research, Department of Cardiology, Oslo University Hospital, Pb 4956 Nydalen, 0424 Oslo, Norway; 2 Faculty of Medicine, University of Oslo, Oslo, Norway; 3 Center for Heart Failure Research, University of Oslo, Oslo, Norway

**Keywords:** P2Y12 receptor antagonists, Residual platelet reactivity, Clopidogrel, Prasugrel, Ticagrelor

## Abstract

**Background:**

Platelet inhibition is important for patients with coronary artery disease. When dual antiplatelet therapy (DAPT) is required, a P2Y_12_-antagonist is usually recommended in addition to standard aspirin therapy. The most used P2Y_12_-antagonists are clopidogrel, prasugrel and ticagrelor. Despite DAPT, some patients experience adverse cardiovascular events, and insufficient platelet inhibition has been suggested as a possible cause. In the present review we have performed a literature search on prevalence, mechanisms and clinical implications of resistance to P2Y_12_ inhibitors.

**Methods:**

The PubMed database was searched for relevant papers and 11 meta-analyses were included. P2Y_12_ resistance is measured by stimulating platelets with ADP ex vivo and the most used assays are vasodilator stimulated phosphoprotein (VASP), Multiplate, VerifyNow (VN) and light transmission aggregometry (LTA).

**Discussion/conclusion:**

The frequency of high platelet reactivity (HPR) during clopidogrel therapy is predicted to be 30%. Genetic polymorphisms and drug-drug interactions are discussed to explain a significant part of this inter-individual variation. HPR during prasugrel and ticagrelor treatment is estimated to be 3–15% and 0–3%, respectively. This lower frequency is explained by less complicated and more efficient generation of the active metabolite compared to clopidogrel. Meta-analyses do show a positive effect of adjusting standard clopidogrel treatment based on platelet function testing. Despite this, personalized therapy is not recommended because no large-scale RCT have shown any clinical benefit. For patients on prasugrel and ticagrelor, platelet function testing is not recommended due to low occurrence of HPR.

## Background

**Fig. 1:**
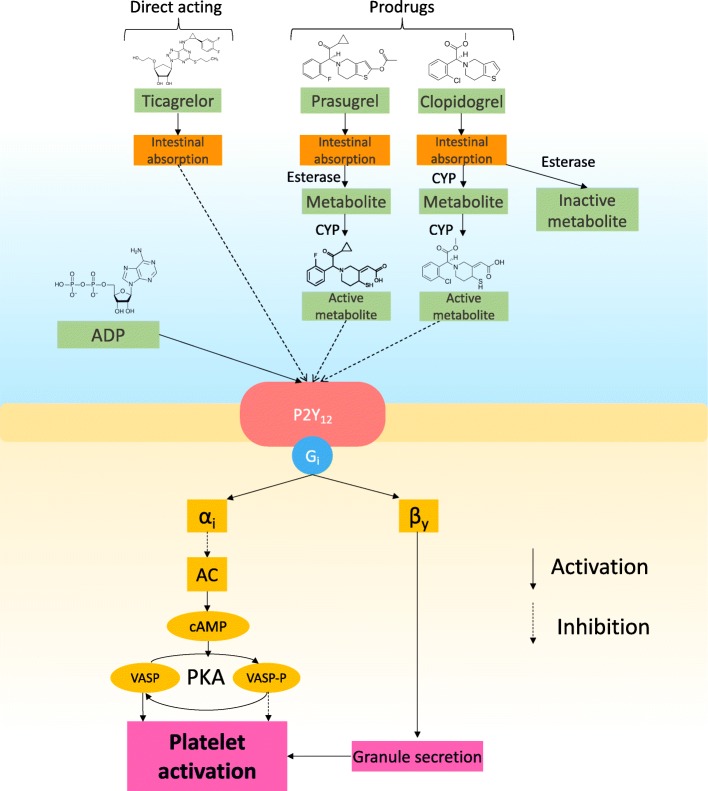
The role of the P2Y_12_ receptors in ADP stimulated platelet activation. Adapted from [1]

Platelet inhibition is pivotal to reduce cardiovascular events (CVE) in patients with coronary artery disease (CAD). The cornerstone in such treatment is aspirin, but when dual antiplatelet treatment (DAPT) is required, adding a P2Y_12_ inhibitor is usually recommended. The most used P2Y_12_ inhibitors are clopidogrel, prasugrel and ticagrelor. Their different properties are shown in Fig. 1 and Table 1.

**Table 1:** Properties of the different P2Y12 inhibitors. Adapted and modified from [2, 3]

	Clopidogrel	Prasugrel	Ticagrelor
Chemical class	Thienopyridine	Thienopyridine	Cyclopentyl-triazolopyrimidine
		
Administration	Oral	Oral	Oral
Dose	300–600 mg orally then 75 mg a day	60 mg orally then 10 mg a day	180 mg orally then 90 mg twice a day
Binding reversibility	Irreversible	Irreversible	Reversible
Binding site	ADP-binding site	ADP-binding site	Allosteric binding site
Activation	Prodrug, with variable liver metabolism	Prodrug, with predictable liver metabolism	Active drug, with additional active metabolite
Onset of loading dose effect	2–6 h	30 min	30 min
Duration of effect	3–10 days	7–10 days	3–5 days
Plasma half-life of active P2Y_12_ inhibitor	30–60 min	30–60 min	6–12 h
Inhibition of adenosine reuptake	No	No	Yes

Clopidogrel has been the most used P2Y_12_ inhibitor in routine clinical practice for years and has been the subject of a considerable amount of research. DAPT with aspirin and clopidogrel was previously the preferred combination, but this changed after prasugrel and ticagrelor were introduced. Prasugrel has replaced clopidogrel in patients with ST-elevation myocardial infarction (STEMI) after percutaneous coronary intervention (PCI), and ticagrelor is preferred in patients with non-ST elevation myocardial infarction (NSTEMI) after PCI [2, 4]. Prasugrel and ticagrelor reduce new cardiovascular events more efficiently in these patient populations, but on the other hand more bleeding complications are reported [5, 6]. After elective PCI in patients with stable CAD, clopidogrel is still the first choice [7].

Despite DAPT, some patients still experience recurrent cardiovascular events. This may be due to many reasons, but insufficient platelet inhibition has been suggested a possible cause, and inter-individual differences in response are well known. A challenge in antiplatelet therapy is the lack of a standardized way to titrate the drug dose to achieve sufficient platelet inhibition and personalize treatment, like we can do with lipid-lowering and blood pressure medication [8].

Lack of response to antiplatelet therapy, termed resistance, non-responsiveness or high platelet reactivity (HPR) despite use of platelet inhibitors, has been widely studied. It has been distinguished between clinical and laboratory non-responsiveness. Clinical non-responsiveness is discussed when platelet-inhibited patients experience cardiovascular events. Laboratory non-responsiveness is defined when platelets still are active ex vivo despite treatment. These phenomena have only to some degree been shown to overlap [8].

Also, non-compliance i.e. patients not taken their medication, has to be considered when discussing the responsiveness/resistance phenomenon in clinical practice. This is, however, not discussed in the present review.

Studies on platelet non-responsiveness were initially focused on aspirin which has been extensively studied. When clopidogrel was introduced, this phenomenon was early addressed, and has later been studied also with regard to other P2Y_12_ inhibitors. The interindividual response variability to clopidogrel is well established [9, 10]. Response variability to ticagrelor and prasugrel, on the other hand, is less known.

The aim of the present work was to summarize the literature on prevalence, mechanisms and clinical implications of resistance to P2Y_12_ receptor inhibitors and give a conclusion based on the reports available.

## Methods

ESC Guidelines on “Ischaemic Heart Disease and Acute Cardiac Care” [2, 11, 12] were used to discuss clinical guidelines for the different states of coronary artery disease.

### Search strategy

Studies until the 11th of December 2017 were included in the literature search. The PubMed database was used. Phrases or synonyms for “P2Y_12_ receptor antagonists” and “drug resistance” (shown below), were used identifying 1228 papers. When limiting the search to English language and last 5 years, in which the novel P2Y_12_ inhibitors have been incorporated into clinical practice, the number of papers was reduced to 540.

Our search strategy was as following:

(“Purinergic P2Y Receptor Antagonists” [mesh] OR ((ADP[Title] OR P2Y12[Title]) AND (Antagonist*[Title] OR blocker*[Title])) OR clopidogrel[Title] OR prasugrel[Title] OR ticagrelor[title]) AND (“Drug Resistance”[Mesh] OR “Pharmacogenetics”[Mesh] OR resistance[Title] OR respons*[Title] OR respond*[Title] OR toleran*[Title] OR nonrespon*[Title] OR reactiv*[Title]) AND “last 5 years”[PDat] AND English[lang]

Further focus on systematic reviews by adding “systematic[sb]” to the search strategy, identified 26 papers. To discover any potential Cochrane reviews, we added “Cochrane Database Syst Rev”[Journal] to the search, but 0 papers were found.

Of the 26 systematic reviews, we excluded 8 due to lack of power, studying non-CAD population or not being meta-analyses.

Results on genetic aspects (7 papers) were excluded from this review due to the complexity without obvious relevance for functionality, other than one specific single nucleotide polymorphisms (SNPs)‘s influence on clopidogrel function. The topic is to some degree featured in the discussion. Thus, 11 meta-analyses are included.

### Methods to determine P2Y_12_ resistance/non-responsiveness

P2Y_12_resistance is measured by stimulating platelets with ADP ex vivo. There are different assays for this purpose and the most used are measure of vasodilator stimulated phosphoprotein (VASP), Multiplate, VerifyNow (VN) and light transmission aggregometry (LTA). Platelet aggregometry induced by ADP is a functional test with a more global aggregation measure than e.g. VASP, which is more specific to drug action at subcellular levels. Aggregometry is the basic principle for VerifyNow, Multiplate and LTA.

Determination of drug response by all these methods have shown to predict clinical outcome in a significant number of patients after PCI [13]. Nevertheless, the expert consensus guidelines do not recommend LTA unless none of the other assays are available. This is due to lack of standardization of this method [13]. The currently recommended assays are therefore the VerifyNow, the Multiplate assay and the VASP assay. However, in clinical practice VerifyNow and Multiplate are preferred due to their standardized and user-friendly set up.

Another issue is determination of cut-off values for the definition of “laboratory non-responsiveness”. The optimal threshold is still being investigated and may vary depending on the clinical situation. The current recommendation is 208 PRU with VerifyNow, 46 AU with the Multiplate assay and 50% with the VASP assay [13].

## Discussion

### Meta-analyses on laboratory non-responsiveness to P2Y12 antagonism

The number of patients included in the analyses investigating laboratory non-responsiveness range from 445 to 5395. This variation may be explained by different inclusion criteria, the number of drugs included and the type and number of laboratory methods used. A summary of the meta-analyses on laboratory non-responsiveness are shown in Table 2.

**Table 2:** Platelet function testing on different antiplatelet therapies and regimens

Authors (year)	Study design	Population	No. studies (no. patients)	Drug and/or intervention	Lab method	Laboratory outcome Data are presented mainly as mean difference in PR with 95% confidence interval (CI) or frequency (%) of HPR
Zhang, H. et al. (2017) [14]	Meta-analysis of RCTs [12] and registry studies [6]	Patients with CAD	16 (2187)	Prasugrel vs. ticagrelor	VN and/or VASP	For the LD, the difference in PR between the prasugrel and ticagrelor groups was [10.80 (− 9.81, 31.40), *p* = 0.30] using the VN test and [− 2.87 (− 6.35, 0.60), *p* = 0.10] using the VASP test. For the MD, the PR was lower in the ticagrelor group than in the prasugrel group, [− 43.37 (− 60.53, − 26.21), *p* < 0.01] using the VN test and [− 9.23 (− 15.82, − 2.64), p < 0.01], using the VASP test.
Lhermusier, T. et al. (2015) [15]	Meta-analysis	Patients with CAD	29 (5395)	Ticagrelor vs. prasugrel vs. clopidogrel	VASP, VN, LTA	Compared with clopidogrel 75 mg, both prasugrel 10 mg and ticagrelor 90 mg × 2 were associated with lower PRU [− 117 (− 134.1, − 100.5)] and [− 159.7 (− 182.6, − 136.6)], respectively), lower PRI [− 24.2 (− 28.2, − 20.3) and [− 33.6 (− 39.9, − 27.6)], respectively), and lower MPA [− 11.8 (− 17, − 6.3) and [− 20.7 (− 28.5, − 12.8)], respectively). Similar results were obtained comparing clopidogrel 75 mg with 150 mg. with prasugrel 10 mg, ticagrelor 90 mg × 2 was associated with lower PRU [− 42.5 (− 62.9, − 21.9)], lower PRI [− 9.3 (− 15.6, − 3.5)], and lower MPA [− 8.9 (− 16.4, − 1.2)].
Lemesle, G. et al. (2015) [16]	Meta-analysis of RCTs [1] and registry studies [6]	Patients with CAD	14 (1822)	Prasugrel vs. ticagrelor	VASP, VN	The frequency of HPR was significantly lower in the ticagrelor group: 1.5% vs. 9.8% (*p* < 0.0001). In studies testing impact of LD, the frequency of HPR was 4.5% (ticagrelor) vs. 13.2% (prasugrel) (*p* = 0.07). In studies testing impact of MD, the frequency was 0.6% (ticagrelor) vs. 7.8% (prasugrel) (p < 0.0001).
Alexopoulos, D. et al. (2014) [17]	Meta-analysis	Patients with CAD	8 (445)	Ticagrelor	VN	Distribution of PR during ticagrelor MD was highly skewed toward lower values. No case of HPR (cut-off ≥230 PRU) was observed. Age and BMI positively affected PR, while current smoking lowered PR.

### Meta-analyses on clinical outcome of non-responsiveness to P2Y12 antagonism

In the analyses investigating clinical outcome the number of patients varies from 605 to 28,178. This wide range may also be explained by different inclusion criteria, the number of drugs included, different study design and follow-up time, in addition to the laboratory methods used. A summary of the meta-analyses on clinical outcome are shown in Table 3.

### Prevalence and mechanisms of high platelet reactivity (HPR) in P2Y_12_-antagonists

#### Clopidogrel

The prevalence of high platelet reactivity (HPR) during clopidogrel treatment is high. However, the estimates have been inconsistent and dependent on the laboratory methods and cut off values used. From the expert consensus guidelines from 2014, the prevalence is predicted to be approximately 30% [13], which also fits with the meta-analysis by D’Ascenzo, F. et al. (Table 3).

**Table 3:** Clinical outcome with different antiplatelet therapies and regimens

Authors (year)	Study design	Population	No. studies (no. patients)	Drug and/or intervention	Lab method	Clinical outcome Risk Ratio (RR) or Odds ratio (OR) with 95% CI are mainly given
Zhou, Y. et al. (2017) [18]	Meta-analysis of RCTs	Patients with CAD undergoing PCI	13 (7290)	CAT vs. IAT based on platelet function testing	VASP, VN, LTA, Multiplate	Testing-guided IAT was associated with a significant reduction in MACE [RR: 0.55 (0.36, 0.84), *p* = 0.005], CV death [RR: 0.60 (0.38–0.96), p = 0.03], ST [RR: 0.58 (0.36, 0.93), *p* = 0.02] and TVR [RR: 0.33 (0.14–0.76), *p* = 0.009] compared to CAT. No significant difference in rate of bleeding events.
Xu, L. et al. (2016) [19]	Meta-analysis of RCTs	Patients with CAD undergoing PCI	13 (5111)	CAT vs. IAT based on platelet function testing	VASP, VN, LTA, Multiplate, TEG	The incidences of CV death, nonfatal MI, and stent thrombosis were significantly lower in the IAT group than in the CAT group [RR: 0.45, (0.36, 0.57), *p* < 0.00001], whereas bleeding was similar between the two groups [RR: 1.05 (0.86, 1.27), *p* = 0.65].
Reny, J. et al. (2016) [20]	Meta-analysis of prospective cohorts and RCTs	Patients with symptomatic atherothrombosis	13 (6478)	Clopidogrel	LTA	The strength of the association between PR and the risk of MACE increased significantly (*p* = 0.04) with the number of risk factors present (age > 75 years, ACS at inclusion, diabetes, and hypertension). No association was detected in patients with no risk factor (*p* = 0.48).
Ma, W. et al. (2015) [21]	Meta-analysis of RCTs	Patients undergoing PCI	17 (4822)	CAT vs. IAT with and without platelet function testing.	VASP, VN, LTA, Multiplate	IAT was generally associated with a significant reduction in the risk of MACE [OR: 0.52 (0.39, 0.71), *p* < 0.0001]. The subgroup with HPR did also benefit from IAT compared to CAT [OR: 0.54 (0.38, 0.77), *p* = 0.0007]. The observed benefits were mainly attributed to treatment-associated reduction in ST [OR: 0.43 (0.23, 0.78), *p* = 0.006] and TVR [OR: 0.38 (0.20, 0.74), *p* = 0.004]. No difference in the rate of major/minor bleeding event between IAT or CAT [OR: 0.80 (0.56, 1.13), *p* = 0.21].
Lin, L. et al. (2015) [22]	Meta-analysis of RCTs	Patients undergoing PCI	8 (3865)	CAT vs. IAT in patients with HPR	VASP, VN, LTA, Multiplate	In patients with HPR, IAT significantly reduced the risk of MACE/MACCE [RR: 0.59 (0.39, 0.88), *p* = 0.01], CV death [RR: 0.33, (0.12, 0.97), *p* = 0.04], ST [RR: 0.43 (0.20, 0.92), *p* = 0.03], and TVR [RR 0.31 (0.10, 0.93), *p* = 0.04], without increasing major bleeding [RR 0.75 (0.43, 1.31), *p* = 0.31] compared with CAT.
D’Ascenzo, F. et al. (2014) [23]	Meta-analysis	Patients with CAD	26 (28178)	Aspirin vs. clopidogrel	VN, LTA, Multiplate, TEG,	HPR was reported in 29% of patients on clopidogrel. HPR was not an independent prognostic indicator of adverse cardiac events in patients with either stable and unstable coronary disease for adverse cardiac events.
Chen, J. et al. (2013) [24]	Meta-analysis	Population with ACS	8 (605)	Clopidogrel with and without PPI	VASP, VN, Multiplate	Compared to clopidogrel treatment alone, patients who received both a PPI and clopidogrel had less of a decrease in the PRI [WMD: 8.18 (6.81, 9.56), *p* < 0.00001], less ADP–induced platelet aggregation inhibition [WMD: 7.28 (2.44, 12.11), *p* = 0.003], higher PRU [WMD: 40.58 (19.31, 61.86), *p* = 0.0002], and higher risks of clopidogrel resistance [OR: 2.49 (1.49, 4.14), *p* = 0.0005]. However, no significant differences for the incidences of MACE were found.

*BMI* body mass index, *CAT* conventional antiplatelet therapy, *CV* cardiovascular, *HPR* high platelet reactivity, *IAT* intensified antiplatelet therapy, *LD* loading dose, *LPR* low platelet reactivity, *LTA* light transmission aggregometry, *MACCE* major adverse cardiac and cerebrovascular events, *MACE* major adverse cardiovascular events, *MI* myocardial infarction, *MD* maintenance dose, *MPA* maximal platelet aggregation, *OR* odds ratio, *PPI* protein pump inhibitors, *PR* platelet reactivity, *PRI* platelet reactivity index, *PRU* platelet reactivity units, *RCTs* randomized controlled trials, *RR* relative risk, *SD* standard dose, *ST* stent thrombosis, *TEG* thrombelastography, *TVR* target vessel revascularization, *VASP* Vasodilator stimulated phosphoprotein, *VN* VerifyNow-P2Y_12_, *WMD* weighted mean differenc

Which factors that cause this huge variation in clopidogrel response is not fully resolved, but the most important factors seem to be genetic polymorphisms and drug-drug interactions [25].

Hepatic activation of clopidogrel and conversion into an active metabolite is essential for the inhibition of the P2Y_12_ receptor [26, 27]. This metabolization is dependent of the cytochrome P450 isoenzymes (CYPs) [28]. The isoenzymes CYP2C19 is shown to be of particular interest and is said to explain 12–15% of the variable response to clopidogrel [10]. About 25 SNPs coding for CYP2C19 have been described in which CYP2C19*2 seems to be of most importance, i.e. shown to reduce serum concentration of the active metabolite and also to reduce inhibition of platelet aggregation [29, 30]. Reduced function of CYP2C19 has been reported to increase the risk for MACE [31, 32].

Drug interactions can also affect clopidogrel response. Rifampicin induces several CYPs, including CYP2C19, and leads to higher levels of active clopidogrel with subsequent greater P2Y_12_ receptor blockade [33]. Ketoconazole on the other hand inhibits CYP3A4 and leads to reduced clopidogrel activation [34]. Proton pump inhibitors (PPI) depend on CYP2C19 metabolism like clopidogrel. Chen et al. have reported that combining these drugs increase the risk of clopidogrel resistance, but may be clinically unimportant, as no significant difference in major adverse cardiac events were observed [24]. Treatment with statins which are metabolized by CYP3A4 has shown not or only slightly to reduce platelet reactivity, but not to affect clinical outcome [35, 36].

Other factors that are discussed to contribute to low clopidogrel response are poor absorption, P2Y12 receptor polymorphisms, increased platelet turnover, different clinical factors like sex, diabetes, kidney disease, obesity, hypercholesterolemia [23, 25, 37].

#### Prasugrel and ticagrelor

There is broad scientific consensus that patients on prasugrel or ticagrelor are less susceptible to HPR than patients on clopidogrel, as also shown from the results in Table 2. Like the estimates for clopidogrel resistance, there has also been discrepancy between the reported prevalence of resistance to both prasugrel and ticagrelor.

The variation in the reported prevalence’s may partly be due to lack of methodological standardization. Difference in the HPR definition across the studies is one limitation [16], but it also seems like PR varies depending on loading sequence, pre-treatment with clopidogrel, time point of testing, switching strategy, and patient population included [38].

Lemesle et al. have published a meta-analysis (Table 2) and included studies looking at the rate of HPR in the acute phase during loading dose (LD), but also during maintenance dose (MD) [16]. When isolating studies that tested PR after loading dose, no significant differences between the ticagrelor and prasugrel group were found. Nevertheless, when testing the impact of the maintenance dose, the rate of HPR was significantly lower in the ticagrelor group. The overall rate of HPR was significantly lower in the ticagrelor vs. prasugrel group [15]. Also the meta-analysis by Zhang et al. describe PR to be similar between the ticagrelor and prasugrel group after loading dose, but lower in the ticagrelor group during maintenance dose [14]. The meta-analysis by Lhermusier et al., though only including studies during maintenance dose, supports this observation [15].

The rate of HPR on prasugrel and ticagrelor treatment has not been established, but it is on maintenance dose estimated to be 3–15% for patients on prasugrel and 0–3% for ticagrelor treated patients [25]. Despite the low PR for both drugs, comparisons have shown that ticagrelor is the most potent platelet inhibitor and has the lowest prevalence of HPR [15, 16, 25].

The differences in HPR between clopidogrel, prasugrel and ticagrelor can partly be explained by the differences in their pharmacokinetics. Prasugrel has more efficient generation of active metabolite compared to clopidogrel [39, 40]. It is less dependent of CYP2C19 metabolism, and therefore not as affected by genetic variants of this enzyme [41, 42]. The most potent agent, ticagrelor, is an active drug and is not dependent on enzyme activation, i.e. is less susceptible to drug-drug interactions or pharmacogenetic influences [43]. Nevertheless, it has been shown that levels of active ticagrelor are affected by genetic variants of SLCO1B1 (solute carrier organic anion transporter family member 1B1) and UGT2B7 (UDP glucuronosyltransferase family 2 member B7). These gene variants have, however, not been shown to have any clinical implication [41]. PR on ticagrelor was affected by age, BMI and smoking status i.e. patients with increasing age and BMI have higher PR, and smokers lower PR [17]. Nevertheless, the PR on ticagrelor was generally very low and the rate of non-responders was 0% in this meta-analysis.

### HPR as a predictor of clinical outcome and personalized antiplatelet therapy

Multiple studies have shown that patients with HPR during clopidogrel treatment are at greater risk for MACE [10]. Because of this, individualization of antiplatelet therapy based on platelet function testing has been studied in several RCTs. The principle in these trials has mainly been to compare the effect of intensified antiplatelet therapy (IAT) against conventional antiplatelet therapy (CAT) on clinical outcome in patients with HPR. The IAT protocols differ in the studies and is either increasing the clopidogrel dose or changing to prasugrel or ticagrelor. The results from these studies are diverging.

A meta-analysis performed by Zhou et al. (Table 3) found that patients undergoing PCI treated with IAT based on platelet function testing had reduced risk of MACE, CV death, stent thrombosis and target vessel revascularization, without any increase in the risk of bleeding [18]. Xu et al. found similar results in their meta-analysis with significantly reduced risk of CV death, nonfatal MI and stent thrombosis in the IAT group [19]. Ma et al. also found that patients with HPR did benefit from IAT compared to conventional antiplatelet therapy (CAT), where the observed benefits were mainly attributed to treatment-associated reduction in stent thrombosis and target vessel revascularization [21]. Even though these meta-analyses reach the same conclusion, they are similar and with some exceptions based on the same studies.

Despite similar results from these three meta-analysis, no large-scale randomized clinical trial has demonstrated any benefit of personalized antiplatelet therapy [37]. The GRAVITAS trial found no difference in clinical endpoints when comparing high dose vs. low dose clopidogrel among patients with HPR undergoing PCI. The number of clinical endpoints in this study was, however, very low, and less than half of the estimated number in the power calculations (5%). In addition, the platelet function testing was undertaken 12–24 h after the PCI, which may be have been too late to affect the outcome [44]. The TRIGGER-PCI study found that switching from clopidogrel to prasugrel in patients with HPR lead to a reduction in platelet reactivity, but no improvement in clinical outcome were observed. However, the trial was stopped prematurely after 6 months due to a lower endpoint rate than expected, and the study did therefore not achieve the desired power. And also in this study, platelet function testing with subsequent adjustment was not done before the morning after PCI [45]. The ARCTIC trial randomly assigned patients to a strategy with platelet function monitoring and treatment adjustment in non-responders, or to standard therapy without monitoring. Of the patients with HPR, about 80% received an increased clopidogrel dose, while only approximately 3% were started on prasugrel. The study showed no significant improvement in clinical endpoints with platelet function testing and subsequent drug adjustment as compared with the conventional strategy [46]. The ANTARCTIC trial randomized patients with acute coronary syndrome (ACS) above 75 years to prasugrel with or without platelet function monitoring with drug adjustment when indicated. They observed no differences in clinical outcome between the two groups [47].

In the meta-analysis by Reny et al. it was reported that the association between the risk of MACE and HPR significantly increases with the number of risk factors [20]. They suggest that the association between MACE and PR is dependent of the patient’s cardiovascular profile. The risk factors that are thought to increase the risk of PR and MACE are among others age > 75, ACS at inclusion, diabetes and hypertension. This is supported by another meta-analysis where HPR did not increase the risk of adverse events after adjusting for risk factors [23]. Lack of multivariate analysis may have confounded the evaluation of the independent risk of HPR and may be the reason why all RCTs have failed when trying to show a beneficial effect of individualized antiplatelet therapy based on platelet function testing. The conflicting results between the meta-analyses and the large RCTs may also be due to publication bias.

### Antiplatelet therapy and platelet function testing in clinical practice

The current guidelines for DAPT is to combine aspirin with a P2Y_12_ blocker. Which P2Y_12_ blocker depends on the clinical situation. For stable CAD patients after elective PCI, DAPT with clopidogrel is recommended, but for patients presenting with ACS prasugrel or ticagrelor are preferred [12].

The ESC guidelines do not recommend platelet function testing in routine clinical practice before or after elective stenting [12]. This is because no large-scale RCT has demonstrated any beneficial effect of adjusting therapy based on platelet function testing during clopidogrel treatment. With regards to prasugrel and ticagrelor, tailored therapy based on platelet function has not been that widely investigated, as HPR on these drugs is rare. Thus, platelet function testing is not recommended in these patients either [13].

The “ACCF/AHA/SCAI Guideline for PCI” also states that platelet function testing should not be used in routine clinical practice. Nevertheless, they say that testing may be considered in patients at high risk for MACE and that alternative agents such as prasugrel and ticagrelor might be considered in clopidogrel-treated patients with HPR [48]. However, these guidelines are from 2011 and are not based on the results from more recent RCTs.

## Conclusion

The prevalence of HPR is greater in patients treated with clopidogrel (approximately 30%) compared to patients on the more novel antiplatelet agents prasugrel (3–15%) and ticagrelor (0–3%). These differences are likely due to different drug pharmacokinetics where prasugrel and ticagrelor have more efficient generation of active metabolite compared to clopidogrel.

Although meta-analyses show an effect of adjusting standard clopidogrel treatment based on platelet function testing, personalized therapy is not recommended because no large-scale RCT have shown any clinical benefit. Nevertheless, it should be noticed that the performed RCTs were underpowered to show any clinical effect. Personalized therapy is neither recommended for patients on prasugrel nor ticagrelor due to low occurrence of HPR on these respective drugs.

## Abbreviations

ACS: acute coronary syndrome
AU: aggregation units
BMI: body mass index
CAD: coronary artery disease
CAT: conventional antiplatelet therapy
CV: cardiovascular
CVE: cardiovascular events
CYPs: cytochrome P450 isoenzymes
DAPT: dual antiplatelet therapy
ESC: European Society of Cardiology
HPR: high platelet reactivity
IAT: intensified antiplatelet therapy
LD: loading dose
LPR: low platelet reactivity
LTA: light transmission aggregometry
MACCE: major adverse cardiac and cerebrovascular events
MACE: major adverse cardiovascular events
MD: maintenance dose
MI: myocardial infarction
MPA: maximal platelet aggregation
NSTEMI: non-ST elevation myocardial infarction
OR: odds ratio
PCI: percutaneous coronary intervention
PPI: protein pump inhibitors
PR: platelet reactivity
PRI: platelet reactivity index
PRU: platelet reactivity units
RCTs: randomized controlled trials
RR: relative risk
SD: standard dose
ST: stent thrombosis
STEMI: ST-elevation myocardial infarction
TEG: thrombelastography
TVR: target vessel revascularization
VASP: Vasodilator stimulated phosphoprotein
VN: VerifyNow-P2Y_12_
WMD: weighted mean difference
